# Cardiac Autonomic Balance Is Altered during the Acute Stress Response in Adolescent Major Depression—Effect of Sex

**DOI:** 10.3390/life13112230

**Published:** 2023-11-20

**Authors:** Ingrid Tonhajzerova, Nikola Ferencova, Igor Ondrejka, Igor Hrtanek, Ivan Farsky, Tomas Kukucka, Zuzana Visnovcova

**Affiliations:** 1 Department of Physiology, Jessenius Faculty of Medicine in Martin, Comenius University in Bratislava, 036 01 Martin, Slovakia; ingrid.tonhajzerova@uniba.sk; 2 Psychiatric Clinic, Jessenius Faculty of Medicine in Martin, Comenius University in Bratislava, University Hospital Martin, 036 01 Martin, Slovakia; igor.ondrejka@uniba.sk (I.O.); igor.hrtanek@uniba.sk (I.H.); ivan.farsky@uniba.sk (I.F.); kukucka17@uniba.sk (T.K.); 3 Biomedical Centre Martin, Jessenius Faculty of Medicine in Martin, Comenius University in Bratislava, 036 01 Martin, Slovakia; 4 Department of Nursing, Jessenius Faculty of Medicine in Martin, Comenius University in Bratislava, 036 01 Martin, Slovakia

**Keywords:** major depressive disorder, adolescent age, cardiac autonomic control, stress response, sex

## Abstract

Autonomic nervous system (ANS) abnormalities are associated with major depressive disorder (MDD) already at adolescent age. The majority of studies so far evaluated parasympathetic and sympathetic branches of ANS individually, although composite indices including cardiac autonomic balance (CAB) and cardiac autonomic regulation (CAR) seem to measure ANS functioning more comprehensively and thus could provide better psychopathologies’ predictors. We aimed to study CAB and CAR derived from high-frequency bands of heart rate variability and left ventricular ejection time during complex stress response (rest–Go/NoGo task–recovery) in MDD adolescents with respect to sex. We examined 85 MDD adolescents (52 girls, age: 15.7 ± 0.14 yrs.) and 80 age- and sex-matched controls. The MDD group showed significantly reduced CAB compared to controls at rest, in response to the Go/NoGo task, and in the recovery phase. Moreover, while depressed boys showed significantly lower CAB at rest and in response to the Go/NoGo task compared to control boys, depressed girls showed no significant differences in evaluated parameters compared to control girls. This study for the first time evaluated CAB and CAR indices in drug-naïve first-episode diagnosed MDD adolescents during complex stress responses, indicating an altered cardiac autonomic pattern (i.e., reciprocal sympathetic dominance associated with parasympathetic underactivity), which was predominant for depressed boys.

## 1. Introduction

Major depressive disorder (MDD) is one of the most serious mental disorders, with an increasing prevalence in adolescents. On the other hand, adolescence represents an important formative phase where physiological, psychosocial, and cognitive changes occur and thus leave adolescents more vulnerable to mental disorders, including MDD [1]. In this aspect, the autonomic nervous system (ANS) plays a key role in modulating emotional, behavioral, and physiological states. Cardiac function is extremely sensitive to autonomic regulatory inputs, whose abnormalities have been associated with major depression already at adolescent age. More specifically, the majority of studies reported reduced cardiac vagal-autonomic modulation associated with depressive symptoms or clinical MDD in children and adolescents [2,3,4,5,6]. Further, our recent study also pointed out that adolescent depressed patients were characterized by cardiac sympathetic overactivity [4]. However, so far, MDD research has focused exclusively on individual cardiac parasympathetic or sympathetic indices. Ongoing evaluation of different contributions of cardiac parasympathetic and sympathetic autonomic measures led to the proposal of the autonomic space model (ASM, [7]), suggesting that heart rate control via parasympathetic nervous system (PNS) and sympathetic nervous system (SNS) activity can vary reciprocally, independently, or coactively, thus providing more comprehensive information on cardiac autonomic outflows and functional effects on the heart [8,9]. More specifically, the conceptualization of cardiac autonomic balance (CAB) includes reciprocal patterns of cardiac autonomic activity (i.e., parasympathetic and sympathetic cardiac activity are negatively correlated), in which the reciprocal sympathetic state is characterized by SNS activation combined with the withdrawal of the PNS activity. On the other hand, a reciprocal parasympathetic state is characterized by PNS activation combined with SNS withdrawal [10]. Moreover, uncorrelated SNS-PNS activity, characterized by uncoupled increases or decreases in SNS or PNS, can occur. Conversely, the conceptualization of cardiac autonomic regulation (CAR) reflects the sum of cardiac autonomic functioning, indicating the overall ANS capacity (i.e., coactivation indicating flexible ANS control in which the PNS responds to a strong SNS response or co-inhibition indicating limited ANS control in which the PNS and SNS provide poor reactivity). In other words, while CAB is defined as the reciprocal balance between cardiac-linked PNS and SNS, CAR is defined as the total activity of both ANS branches [9,11]. To sum up, CAB and CAR can represent promising indices of cardiac autonomic functioning that indicate overall autonomic flexibility and adaptability [12].

Traditionally, both CAB and CAR are calculated using the analysis of heart rate variability in the high-frequency band (HF-HRV), reflecting respiratory sinus arrhythmia as an index of cardiovagal autonomic modulation, and systolic time interval pre-ejection period (PEP) as a measure of cardiac sympathetic inotropy [11,12]. However, the calculation of CAB and CAR should reflect the influence of cardiac chronotropy on both parasympathetic and sympathetic measures [9]. In this way, the left ventricular ejection time (LVET), as a systolic time interval reflecting the sympathetic chronotropic effect, is considered a superior measure for calculating CAB and CAR instead of PEP [9]. 

To date, only two studies have explored CAB and CAR in association with depression. While resting values of CAB were lower in young adults with current major depression [13], increased CAB was reported during the application of the different stressors in youth with a history of juvenile-onset depression [12]. In this line, the determination of cardiac autonomic control using composite metrics appears to be important in MDD, particularly during the complex stress response characterized by three phases: rest, reactivity, and recovery [14]. From this point of view, stressful events are characterized by distinct temporal dynamics: resting before the stressful event, reactivity during the stressful event, and recovery after the stressful event [14]. In this context, various aspects of these temporal stress dynamics can be investigated, such as the physiological stress complex response [15]. More specifically, the reactivity to stress represents the change between baseline and completing a specific task (e.g., physical, emotional, or cognitive) [14]. One of the specific cognitive tasks represents the Go/NoGo task, designed as a neuropsychological test focused on executive functions such as response inhibition [16]. The Go/NoGo task as a mental stressor alters the balance between sympathetic and parasympathetic nervous system activity; however, the detailed analysis of complex autonomic neural activity in response to the Go/NoGo task is rare [17]. Moreover, cardiac autonomic responses to stress can differ between males and females [18,19]. However, whether the effect of depression on the cardiac autonomic regulatory capacity differs as a function of sex needs to be resolved. 

Therefore, our study has focused on two main goals. Firstly, we aimed to analyze potential differences in cardiac autonomic functioning between adolescent drug-näive, first-episode diagnosed MDD patients and healthy controls during complex stress response using: (1) composite indices CAB and CAR reflecting parasympathetic-sympathetic coupling calculated from HF-HRV and LVET; (2) individual indices reflecting cardiac vagally-mediated autonomic modulation (i.e., HF-HRV and baroreflex sensitivity (BRS)); and indices reflecting cardiac sympathetically-mediated beta-adrenergic autonomic modulation (i.e., LVET and high-frequency band of systolic blood pressure variability (HF-SBPV)). Secondly, we aimed to study the effect of sex on the cardiac autonomic patterns between adolescent patients with major depression and healthy adolescents. To our best knowledge, this is the first study evaluating the MDD and sex-dependent impact on cardiac parasympathetic-sympathetic coupling along the ASM at adolescent age. 

## 2. Materials and Methods

### 2.1. Subjects

**Figure 1:**
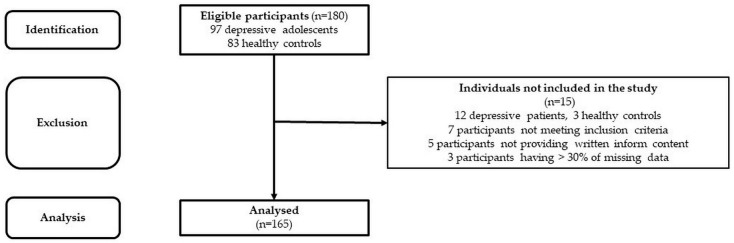
STROBE flow chart of the participants.

The studied cohort consists of 85 adolescent patients suffering from MDD (average age: 15.7 ± 0.14 yrs., 52 girls–average age: 15.6 ± 0.18 yrs.; 33 boys–average age: 15.9 ± 0.2 yrs.) and 80 healthy subjects matched for sex (assigned at birth) and age (control group–average age: 15.9 ± 0.15 yrs., 51 girls–average age: 16.0 ± 0.19 yrs.; 29 boys–average age: 15.9 ± 0.27 yrs.) (see Figure 1). The MDD patients included in this study were recruited from the inpatients admitted to the Psychiatric Clinic of the Jessenius Faculty of Medicine and University Hospital in Martin. For the diagnosis of severe single-episode depression without psychotic symptoms and other comorbid mental disorders, an unstructured diagnostic interview was used as a clinical investigation by a child/adolescent psychiatrist according to the Diagnostic and Statistical Manual of Mental Disorders (DSM-5, [20]). The examination of MDD patients was performed before pharmacotherapy within the first days of hospitalization in the psychiatric clinic. The inclusion criteria for MDD patients were the following: (1) the MDD diagnosis according to DSM-5 [20], (2) the adolescent age period from 10 to 19 years according to WHO [21], (3) a severe depressive episode without psychotic symptoms, and (4) no pharmacotherapy. The exclusion criteria for MDD and control groups were the following: history of neurological, metabolic, endocrine, respiratory, and cardiovascular diseases, acute infection, and abnormal weight (underweight, overweight, or obesity). Moreover, the subjects in the control group have never been treated for any psychiatric disorders. Next, all participants were instructed to refrain from substances influencing the activity of the cardiovascular system for at least 12 h before the examination (e.g., caffeine, drugs, etc.). The study was approved by the Ethics Committee of the Jessenius Faculty of Medicine in Martin, Comenius University in Bratislava (protocol code EK1970/2017). All subjects and their legal representatives were thoroughly instructed about the study protocol and confirmed their participation by written informed consent prior to examination.

### 2.2. Study Protocol

The participants were examined in the psychophysiological laboratory (Biomedical Centre Martin, Psychiatric Clinic, Jessenius Faculty of Medicine in Martin) under standard conditions (a quiet room, a temperature of 23 °C, humidity around 50%, minimization of stimuli) in the morning between 9:00 and 11:00 a.m. after a light breakfast at least 2 h before the examination. Firstly, the anthropometric body analysis was performed using the multi-segmental and multi-frequency (20/100 kHz) bioimpedance device InBody 120 (Biospace Co., Ltd., Seoul, Republic of Korea). The body mass index (BMI) values were compared to age- and sex-specific BMI cut-offs, which correspond to the adult BMI range between 18.5 and 25 kg/m^2^ for normal weight and the threshold of 30 kg/m^2^ for obesity [22]. Subsequently, only subjects without weight abnormalities were included in the study.

**Figure 2:**
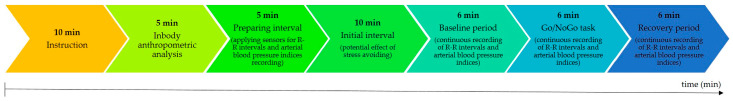
Time schedule of the study protocol.

Next, the participants were comfortably seated in a special armchair. The sensors for continuous beat-to-beat recordings of the R-R intervals with a sampling frequency of 1000 Hz (Polar V800, Polar Electro, Kempele, Finland) and of the blood pressure signal using finger cuff methods with a sampling frequency of 200 Hz (Finometer Midi Model II, Finapress Medical System, Amsterdam, The Netherlands) were applied. In order to minimize the influence of hydrostatic pressure on the finger cuff pressure, a built-in height-correction system was used, which allowed the pressure in the brachial artery to be reconstructed. Further, a relaxation period lasting 10 min was used to avoid the potential effects of stress, followed by a stress protocol consisting of three periods: rest, a stress period (Go/NoGo task), and after stress (recovery). Each period of the stress protocol lasted for 6 min (see Figure 2).

### 2.3. Depressive Symptoms Assessment

Finally, all participants completed the children’s depression inventory (CDI) questionnaire, which investigates the presence of depressive symptoms in the previous two weeks [23]. Specifically, the CDI questionnaire consists of 27 questions (each with three options of answer) scored on a scale of 0 (symptom absence) to 2 (definite symptoms) to assess the severity of depression symptoms in children and adolescents. A total score is evaluated as the sum of all items. The range is from 0 to 54 points, whereas higher scores indicate greater depression severity [23].

### 2.4. Go/NoGo Task

The neuropsychological Go/NoGo task was used as a stress stimulus (FlexComp Infinity, BioGraph Infinity Software Ver 6.8 Update, Thought Technology Ltd., Montreal, QU, Canada). In this version, the red letter X (NoGo stimulus) or green circle (Go stimulus) randomly appeared in the center of the computer on the monitor for a different brief period (the most often appearing period was 0.5 s, the interval between consecutive stimuli random in the range from 1.5 s to 3 s). The principle of the Go/NoGo task is pressing the special button by the subject as soon as possible when the Go stimulus (green circle) appears on a computer monitor and not responding to the NoGo stimulus (red letter X) on a computer monitor [24,25].

### 2.5. Evaluated Parameters

#### 2.5.1. Cardiac Vagal Autonomic Modulation

HF-HRV:

Before the analysis, all continuous R-R interval recordings were carefully checked, and artifacts were removed manually. The five-minute artifact-free R-R interval time series was used for conventional (spectral) analysis. The spectral-domain analysis was assessed by resampling of R-R interval time series using cubic spline interpolation with a rate of 4 Hz and by detrending through a smoothing parameter Λ = 500 [26]. Following, spectral power in HF-HRV (0.15–0.40 Hz) was analyzed by an autoregressive model with a Burg periodogram [27,28]. The HF-HRV is considered a cardiac vagal autonomic modulation index [29,30,31]. As the HF-HRV variable was not normally distributed and displayed a high inter-individual difference/variability, it was logarithmically transformed according to the recommendations for psychophysiological research [29]. After logarithmic transformations, the HF-HRV data were normally distributed.

BRS:

BRS (ms/mmHg) was calculated from continuous beat-to-beat arterial pressure waveform recordings using the sequential cross-correlation method [32]. BRS represents changes in the interbeat interval (ms) for a simultaneously occurring difference in blood pressure (BP, mmHg) and the sensitivity of vagally-mediated heart rate upon BP deviations [33,34] and is widely used to quantify the vagal component of the reflex [35].

#### 2.5.2. Cardiac Beta-Adrenergic Sympathetic Autonomic Modulation

LVET:

Before the analysis, all continuous blood pressure recordings were carefully checked, and artifacts were removed manually. Then, the recordings were analyzed by BeatScope Easy software BeatScope^®^ Easy V2 (Finapres Medical System, Amsterdam, The Netherlands). From a physiological aspect, the LVET represents the time interval from aortic valve opening to aortic valve closure, reflecting the duration of the left ventricle to eject blood to the aorta and indicating cardiac sympathetic chronotropic influence [9,36]. The index LVET (ms) was evaluated as the time between the current upstroke and the dicrotic notch (BeatScope Easy software).

HF-SBPV:

Beat-to-beat systolic blood pressure (SBP, mmHg) and diastolic blood pressure (DBP, mmHg) were monitored through the finger cuff with a sampling rate of 200 Hz by Finometer MIDI Model II and processed using Beat-Scope Easy software (Finapres Medical System, The Netherlands). Five-minute artifact-free recordings were resampled by cubic spline interpolation at a frequency of 2 Hz. The high frequency band of the systolic blood pressure variability (HF-SBPV, 0.15–0.40 Hz) was analyzed using a fast Fourier transform with a window width of 128 samples and 50% overlapping. Similar to the HF-HRV, the HF-SBPV was not normally distributed and displayed high inter-individual difference/variability; it was logarithmically transformed. After logarithmic transformations, the HF-SBPV data were normally distributed. HF-SBPV represents an index of the sympathetically β-adrenoreceptor-mediated modulation of cardiac activity [37]. 

#### 2.5.3. Cardiac Autonomic Balance and Cardiac Autonomic Regulation

**Figure 3:**
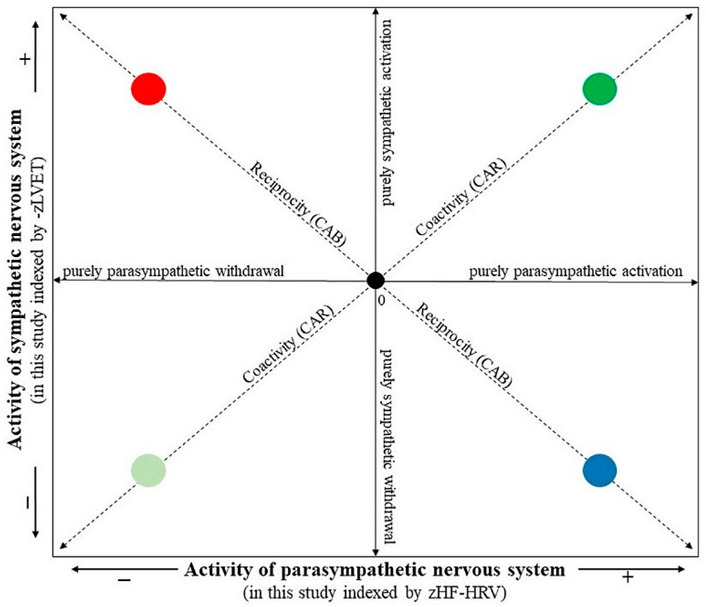
The two-dimensional representation of the autonomic space (according to [10]). The • 0 intersection illustrates the mean position along parasympathetic and sympathetic dimensions. The reciprocity diagonal represents a dimension of reciprocally controlled autonomic divisions. Individuals in the reciprocal parasympathetic quadrant would have relatively high CAB scores, while individuals in the reciprocal sympathetic quadrant would have relatively low CAB scores. The coactivity diagonal represents the regulation dimension indexing the total autonomic activity. Individuals in the coactivation quadrant would have relatively high CAR scores, while individuals in the co-inhibition quadrant would have relatively low CAR scores. The location along the parasympathetic and sympathetic axes represents patterns of independent parasympathetic and sympathetic control. The individual quadrants represent • reciprocal parasympathetic, • reciprocal sympathetic, • co-activation and • co-inhibition modes.

The composite indices, CAB and CAR, were calculated from HF-HRV and LVET parameters. In order to combine the different measurement scales of HF-HRV and LVET into a single index of CAB and CAR, each variable was standardized by transforming raw data to z scores using the formula z = (x − M)/SD, where z = standardized score, x = proband’s raw data, M = mean of the combined group (i.e., depressive and control groups together), and SD = standard deviation of the combined group. As greater sympathetic activity is associated with a shortened LVET, the LVET was first multiplied by −1 for ease in interpreting values (i.e., higher –zLVET indicates higher sympathetic activity, similarly, higher zHF-HRV indicates higher parasympathetic activity). The CAB index was derived as the difference between zHF-HRV and –zLVET (i.e., CAB = zHF-HRV − (–zLVET)). A higher CAB indicates reciprocal parasympathetic control, whereas a lower CAB reflects reciprocal sympathetic control. The CAR index was derived as a summation of zHF-HRV and –zLVET (i.e., CAR = zHF-HRV + (−zLVET)). A higher CAR reflects coactivation, and a lower CAR reflects coinhibition of both autonomic branches [9,11]. In this way, a two-dimensional autonomic space reflecting SNS-PNS reciprocal activity and SNS-PNS coactivation/coinhibiton is presented in Figure 3 (according to [10]).

### 2.6. Statistical Analysis

The data were explored and analyzed in jamovi version 1.2.27 (Sydney, Australia). Data distributions (Gaussian/non-Gaussian) were evaluated using the Shapiro–Wilk normality test. The HF-HRV and HF-SBPV were logarithmically transformed because of high inter-individual differences. After logarithmic transformations, the HF-HRV and HF-SBPV were normally distributed. The analysis of variance (ANOVA, jamovi version 1.2.27) with two fixed factors (group and sex) was used for basic group characteristics with the Bonferoni post hoc test. The repeated measures ANOVA with three fixed factors (group, sex, and period) was used for all evaluated parameters, with the Bonferoni post hoc test controlling the false discovery rate as well as the family rise error rate for the evaluated data. Size effect estimations by Cohen’s d were applied [38,39]. In addition, a priori power analysis was used to determine the required sample sizes, indicating at least 21 boys and 42 girls per group to reliably detect group and sex differences. Further, the associations between depressive scores indexed by CDI, CAR, and CAB during all periods of the examined protocol were analyzed using Spearman’s rank-order correlation test. Data were expressed as mean ± SEM. The results are considered statistically significant if *p* < 0.05. 

## 3. Results

### 3.1. Basic Characteristics

The basic characteristics of MDD patients and control participants are summarized in Table 1. The statistical analysis ANOVA revealed a significant effect of group for parameters BMI and CDI (F[1] = 7.38, *p* = 0.007; F[1] = 290.64, *p* < 0.001, respectively). Post-hoc analysis showed significantly increased BMI in the MDD group compared to the control group (*p* = 0.007, Cohen’s d = −0.437). Further, CDI was significantly increased in the whole MDD group compared to controls (*p* < 0.001, Cohen’s d = 2.850), as well as in MDD girls compared to control girls (*p* < 0.001, Cohen’s d = 2.535) and MDD boys compared to controls (*p* < 0.001, Cohen’s d = 3.174). No significant changes were found in other parameters.

### 3.2. Evaluated Parameters during Stress Protocol

The repeated measures ANOVA revealed significant effect of group for parameters R-R intervals, lnHF-SBPV, BRS, zHF-HRV, –zLVET, CAB, and CAR (F[1] = 33.39, *p* < 0.001; F[1] = 9.96, *p* = 0.002; F[1] = 29.79, *p* < 0.001; F[1] = 12.50, *p* < 0.001; F[1] = 28.77, *p* < 0.001; F[1] = 35.12, *p* < 0.001; F[1] = 4.53, *p* = 0.035, respectively); significant effect of sex for parameters –zLVET and CAB (F[1] = 8.77, *p* = 0.004; F[1] = 6.84, *p* = 0.010, respectively); and significant effect of period for parameters R-R intervals, SBP, DBP, lnHF-SBPV, and BRS (F[2] = 3.42, *p* = 0.034; F[2] = 158.40, *p* < 0.001; F[2] = 104.92, *p* < 0.001; F[2] = 11059.42, *p* < 0.001; F[1] = 17.43, *p* < 0.001, respectively). Moreover, a significant effect of the group x sex interaction was found for the parameters SBP and DBP (F[1] = 4.92, *p* = 0.028; F[1] = 5.59, *p* = 0.019); a significant effect of the group x period interaction for the parameters zHF-HRV and CAR (F[2] = 10.46, *p* < 0.001; F[2] = 7.38, *p* < 0.001, respectively); and a significant effect of the sex x period interaction for the parameters R-R intervals, lnHF-SBPV, and CAR (F[2] = 6.14, *p* = 0.002; F[2] = 5.39, *p* = 0.005; F[2] = 3.94, *p* = 0.020, respectively). No significant effect was found for the group × sex × period interaction for all evaluated parameters.

#### 3.2.1. Between-Group Comparison during Baseline Period

##### MDD Patients vs. Control Probands

Parameters R-R intervals and LVET were significantly shortened in MDD patients compared to controls (*p* < 0.001, Cohen’s d = 0.363; *p* < 0.001, Cohen’s d = −0.917, respectively). Further, BRS, zHF-HRV, and CAB were significantly decreased in MDD patients compared to controls (*p* < 0.001, Cohen’s d = −0.790; *p* < 0.001, Cohen’s d = −0.901; *p* < 0.001, Cohen’s d = −0.823, respectively). Parameter –zLVET was significantly increased in MDD patients compared to controls (*p* = 0.032, Cohen’s d = −0.482). No significant change was found in the remaining parameters between MDD and the control group.

##### MDD Boys vs. Control Boys

Parameters R-R intervals and LVET were significantly shortened in MDD patients compared to controls (*p* = 0.010, Cohen’s d = 0.344; *p* = 0.006, Cohen’s d = −0.954, respectively). Parameters zHF-HRV and CAB were significantly lower in depressive boys compared to controls (*p* = 0.017, Cohen’s d = −1.706; *p* = 0.005, Cohen’s d = −1.004, respectively). No significant changes were found in the remaining parameters.

##### MDD Girls vs. Control Girls

Parameters R-R intervals and LVET were significantly shortened in depressive girls compared to controls (*p* < 0.001, Cohen’s d = 0.382; *p* < 0.001, Cohen’s d = −0.880, respectively). Moreover, lnHF-SBPV was significantly increased and BRS was significantly decreased in MDD girls compared to the control group (*p* = 0.005, Cohen’s d = 0.636; *p* = 0.011, Cohen’s d = −0.774, respectively). No significant changes were found in the remaining parameters.

**Figure 4:**
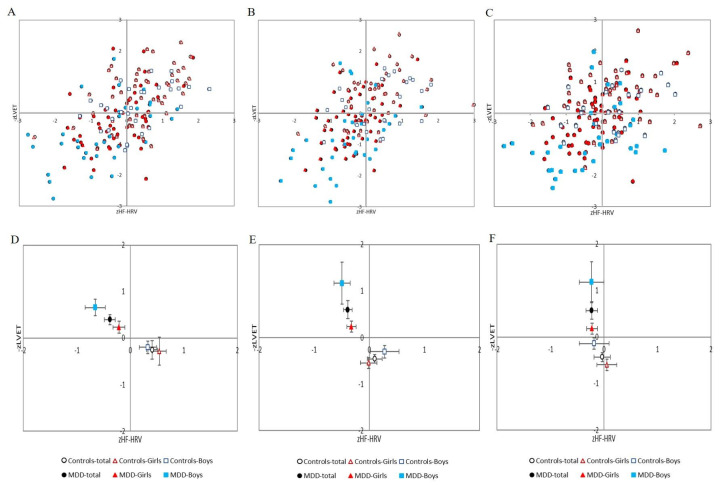
(**A**–**C**) Distribution of cardiac beta-adrenergic sympathetic activity indexed by z score of left ventricular ejection time (−zLVET) and cardiac parasympathetic activity indexed by z score of spectral power in the high-frequency band of the heart rate variability (zHF-HRV) scores into autonomic space in MDD and control groups with respect to sex: (**A**) at rest; (**B**) during Go/NoGo task; (**C**) in recovery period (i.e., after Go/NoGo task). (**D**–**F**) Graphical representation of the parameters −zLVET and zHF-HRV (expressed as mean and SEM) into autonomic space across the MDD and control groups: (**D**) at rest; (**E**) during Go/NoGo task; (**F**) in recovery phase (i.e., after Go/NoGo task). MDD-total—full black circle, MDD-Boys—full blue squares, MDD-Girls—full red triangles, Controls-total—empty black circle, Controls-Boys—empty blue squares, and Controls-Girls—empty red triangles. MDD—major depressive disorder.

**Figure 5:**
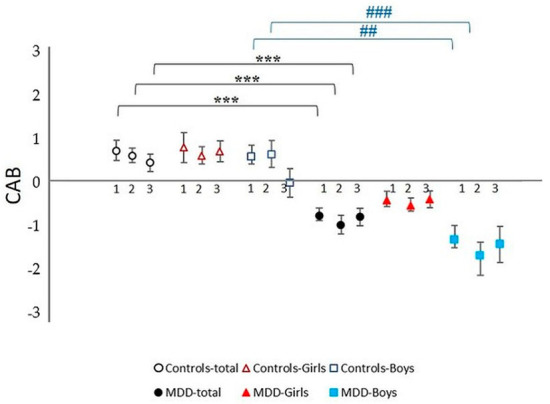
Cardiac autonomic balance (CAB) during complex stress protocol in MDD and control groups with respect to sex: 1—rest; 2—Go/NoGo task, 3—recovery period after Go/NoGo task; MDD-total—full black circle, MDD-Boys—full blue squares, MDD-Girls—full red triangles, Controls-total—empty black circle, Controls-Boys—empty blue squares, and Controls-Girls—empty red triangles; *** represents statistically significant differences in total MDD vs. total control group at level *p* < 0.001, ## represents statistically significant differences in MDD Boys group vs. control Boys group at level *p* < 0.01; ### represents statistically significant differences in MDD Boys group vs. control Boys group at level *p* < 0.001. MDD—major depressive disorder.

Additionally, post-hoc analysis revealed no significant sex differences in the MDD group alone (MDD girls vs. MDD boys) or in the control group (control girls vs. control boys). All results are summarized in Table 2, Figure 4A–F and Figure 5.

#### 3.2.2. Between-Group Comparison during Go/NoGo Task

##### MDD Patients vs. Control Probands

Parameters R-R intervals and LVET were significantly shortened in MDD patients compared to controls (*p* < 0.001, Cohen’s d = −0.792; *p* < 0.001, Cohen’s d = −1.050). Parameters lnHF-HRV, BRS, zHF-HRV, and CAB were significantly decreased in the depressive group compared to controls (*p* < 0.001, Cohen’s d = −0.310; *p* < 0.001, Cohen’s d = −0.847; *p* < 0.001, Cohen’s d = −0.554; *p* < 0.001, Cohen’s d = −0.910, respectively). Furthermore, lnHF-SBPV and –zLVET were significantly increased in depressive patients compared to controls (*p* = 0.020, Cohen’s d = 0.541; *p* < 0.001, Cohen’s d = −0.823, respectively). No significant changes were found in the remaining parameters.

##### MDD Boys vs. Control Boys

The mean R-R intervals and LVET were significantly shortened in MDD boys compared to controls (*p* < 0.001, Cohen’s d = −0.947; *p* < 0.001, Cohen’s d = −1.211, respectively). The lnHF-HRV, BRS, and CAB were significantly decreased in MDD boys compared to controls (*p* < 0.001, Cohen’s d = −0.424; *p* = 0.012, Cohen’s d = −0.904; *p* < 0.001, Cohen’s d = −1.222, respectively). Moreover, –zLVET was significantly higher in MDD boys than control boys (*p* = 0.002, Cohen’s d = −1.078). No significant changes were found in the remaining parameters.

##### MDD Girls vs. Control Girls

The mean R-R intervals and LVET were significantly shortened in MDD girls compared to controls (*p* = 0.035, Cohen’s d = −0.636; *p* = 0.003, Cohen’s d = −0.885, respectively). The lnHF-HRV and BRS were significantly decreased in MDD compared to control girls (*p* = 0.010, Cohen’s d = −0.195; *p* = 0.004, Cohen’s d = −0.791, respectively). Further, lnHF-SBPV was significantly higher in depressive girls than controls (*p* = 0.001, Cohen’s d = 0.709). No significant changes were found in the remaining parameters.

In the stress period, post-hoc analysis revealed no significant sex differences in the MDD group alone (MDD girls vs. MDD boys) or in the control group (control girls vs. control boys). All results are summarized in Table 2, Figure 4A–F and Figure 5.

#### 3.2.3. Between-Group Comparison during Recovery Period

##### MDD Patients vs. Control Probands

The mean R-R intervals and LVET were significantly shortened in MDD patients compared to controls (*p* < 0.001, Cohen’s d = −0.767; *p* < 0.001, Cohen’s d = −0.970, respectively). The lnHF-HRV, BRS, and CAB were significantly decreased, and parameters –zLVET and CAR were significantly increased in MDD compared to the control group (*p* = 0.014, Cohen’s d = −0.493; *p* < 0.001, Cohen’s d = −1.020; *p* < 0.001, Cohen’s d = −0.702; *p* < 0.001, Cohen’s d = −0.782; *p* = 0.011, Cohen’s d = 0.472, respectively). No significant changes were found in the remaining parameters.

##### MDD Boys vs. Control Boys

The LVET was significantly shortened in MDD boys compared to controls (*p* = 0.004, Cohen’s d = −1.051). Parameter –zLVET was significantly higher and parameter BRS was significantly lower in MDD boys than controls (*p* = 0.013, Cohen’s d = −0.983; *p* = 0.028, Cohen’s d = −1.149, respectively). No significant changes were found in the remaining parameters.

##### MDD Girls vs. Control Girls

The LVET was significantly shortened in MDD girls compared to controls (*p* = 0.003, Cohen’s d = −0.889). The lnHF-SBPV was significantly increased and the BRS was significantly decreased in MDD girls than controls (*p* = 0.033, Cohen’s d = 0.534; *p* = 0.045, Cohen’s d = −0.884, respectively). No significant changes were found in the remaining parameters.

Moreover, parameter lnHF-SBPV was significantly increased in control boys compared to control girls (*p* = 0.009, Cohen’s d = −0.662). No significant sex differences were found in the remaining parameters. All results are summarized in Table 2, Figure 4A–F and Figure 5.

#### 3.2.4. Comparison of the Individual Periods of the Protocol (Baseline vs. Go/NoGo Task vs. Recovery Period) within MDD and Control Groups

##### MDD Group

Parameters lnHF-HRV, SBP, DBP, and lnHF-SBPV were significantly increased during the Go/NoGo task and recovery period compared to the baseline period (*p* < 0.001 for all). Parameter SBP was significantly decreased during the recovery period compared to the Go/NoGo task (*p* < 0.001). The lnHF-SBPV was significantly increased during the recovery period compared to the Go/NoGo task (*p* < 0.001), and the BRS was significantly higher during the Go/NoGo task compared to the baseline period (*p* = 0.037). No significant changes were found in the remaining evaluated parameters between the individual evaluated periods in the MDD group.

##### MDD Boys

Parameters lnHF-HRV, SBP, DBP, and lnHF-SBPV were significantly increased during the Go/NoGo task and recovery period compared to the baseline period (*p* < 0.001; *p* < 0.001; *p* < 0.001; *p* = 0.011; *p* < 0.001; *p* = 0.002; *p* < 0.001; *p* < 0.001, respectively). The SBP was significantly decreased and the lnHF-SBPV was significantly increased during the recovery period compared to the Go/NoGo task (*p* < 0.001 for both). No significant changes were found in the remaining evaluated parameters between the individual evaluated periods in MDD boys.

##### MDD Girls

Parameters lnHF-HRV, SBP, DBP, and lnHF-SBPV were significantly increased during the Go/NoGo task and recovery period compared to the baseline period (*p* < 0.001 for all). Further, the lnHF-SBPV was significantly increased during the recovery period compared to the Go/NoGo task (*p* = 0.030). No significant changes were found in the remaining evaluated parameters.

##### Control Group

Parameters lnHF-HRV, SBP, DBP, and lnHF-SBPV were significantly increased during the Go/NoGo task and recovery period compared to baseline (*p* < 0.001 for all). Parameters lnHF-HRV, SBP, and DBP were significantly decreased during the recovery period compared to the Go/NoGo task (*p* < 0.001 for all). The LVET was significantly shortened, and the lnHF-SBPV was significantly increased during the recovery period compared to the Go/NoGo task (*p* = 0.019, *p* < 0.001, respectively). Further, parameter BRS was significantly higher during the Go/NoGo task compared to baseline and significantly lower during recovery compared to the Go/NoGo task (*p* < 0.001 for both). Lastly, the statistical analysis revealed significantly decreased zHF-HRV and CAR during the recovery period compared to the baseline period (*p* < 0.001; *p* = 0.037, respectively). No significant changes were found in the remaining parameters between the individual evaluated periods.

##### Control Boys

Parameters lnHF-HRV, SBP, DBP, and lnHF-SBPV were significantly increased during the Go/NoGo task as well as the recovery period compared to the baseline period (*p* < 0.001; *p* < 0.001; *p* = 0.006; *p* < 0.001; *p* < 0.001; *p* < 0.001; *p* = 0.023; *p* < 0.001, respectively). The mean R-R intervals and LVET were significantly shortened, the mean SBP and DBP were significantly decreased, and the lnHF-HRV and lnHF-SBPV were significantly increased during the recovery period compared to the Go/NoGo task (*p* = 0.011; *p* = 0.031; *p* < 0.001; *p* = 0.011; *p* < 0.001; *p* < 0.001, respectively). Further, the BRS was significantly higher during the Go/NoGo task compared to the baseline period (*p* = 0.031) and significantly lower during the recovery period compared to the Go/NoGo task (*p* = 0.039). No significant changes were found in the remaining parameters.

##### Control Girls

Parameters lnHF-HRV, SBP, DBP, and lnHF-SBPV were significantly increased during the Go/NoGo task and recovery period compared to the baseline period (*p* < 0.001; *p* < 0.001; *p* < 0.001; *p* < 0.001; *p* < 0.001; *p* < 0.001; *p* = 0.008; *p* < 0.001, respectively). The mean SBP and DBP were significantly decreased, and the lnHF-SBPV was significantly increased during the recovery period compared to the Go/NoGo task (*p* < 0.001; *p* = 0.002; *p* = 0.028, respectively). Statistical analysis revealed significantly decreased zHF-HRV during the Go/NoGo task compared to baseline (*p* = 0.018) as well as during the recovery period compared to baseline (*p* = 0.039). No significant changes were found in the remaining parameters between the individual evaluated periods.

### 3.3. Correlation Analysis between Total Score of CDI and Cardiac Composite Indices (CAB and CAR)

#### 3.3.1. Correlation Analysis for Whole Group

**Figure 6:**
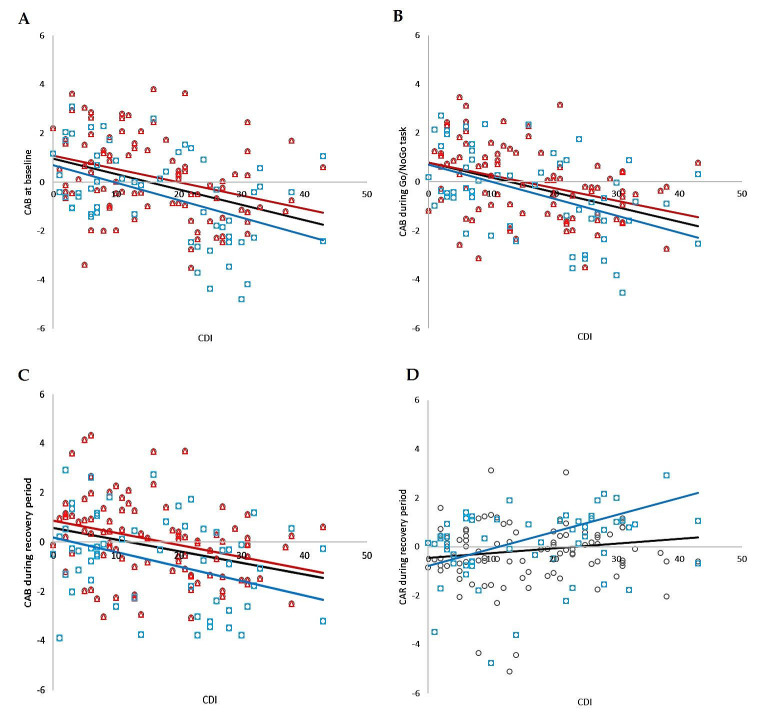
The correlation analysis of total CDI score and CAB in total group (i.e., MDD and control group together; black circles, black line), individually in boys group (blue squares, blue line) and individually in girls group (red triangles, red line); (**A**) at baseline, (**B**) during Go/NoGo task, (**C**) during recovery, (**D**) correlation analysis of total CDI score and CAR during recovery in total group (circles, black line) and individually in boys group (blue squares, blue line). CDI—Child’s Depression Inventory; CAB—cardiac autonomic balance; CAR—cardiac autonomic regulation.

Correlation analysis revealed significant negative relationships between CDI and CAB at baseline, during the Go/NoGo task, and during the recovery period, as well as significant positive relations between CDI and CAR during recovery in the whole group (r = −0.382, *p* < 0.001; r = −0.429, *p* < 0.001; r = −0.320, *p* < 0.001; r = 0.169, *p* = 0.039, respectively). No significant correlations were found between CDI and CAR at baseline or between CDI and CAR during the Go/NoGo task. The correlation analysis is summarized in Figure 6.

#### 3.3.2. Correlation Analysis for Boys

Correlation analysis revealed significant negative relationships between CDI and CAB at baseline, during the Go/NoGo task, and during the recovery period, as well as significant positive relations between CDI and CAR during recovery in the boy group (r = −0.451, *p* < 0.001; r = −0.504, *p* < 0.001; r = −0.276, *p* = 0.036; r = 0.337, *p* = 0.010, respectively). No significant correlations were found between CDI and CAR at baseline or between CDI and CAR during the Go/NoGo task. The correlation analysis is summarized in Figure 6.

#### 3.3.3. Correlation Analysis for Girls

Correlation analysis revealed significant negative relationships between CDI and CAB at baseline, during the Go/NoGo task, and during the recovery period in the girls group (r = −0.350, *p* < 0.001; r = −0.399, *p* < 0.001; r = −0.361, *p* = 0.036, respectively). No significant correlations were found between CDI and CAR in all periods of the stress protocol. The correlation analysis is summarized in Figure 6.

## 4. Discussion

This study, for the first time, explored cardiac parasympathetic and sympathetic coupling in a more comprehensive view of autonomic regulatory functioning during complex stress responses in first-episode-diagnosed current depression at adolescent age with respect to sex. Flexible and adaptive ANS functioning is a critical modulator of physiological and emotional processes implicated in psychological health. Conversely, abnormal ANS functioning characterized by sympathetic dominance and parasympathetic underactivity is commonly observed in psychopathology, including MDD. Importantly, it has been proposed that composite indices represent more sensitive measures of ANS functioning compared to separate PNS and/or SNS indices [40]. Given that the autonomic space model incorporates both PNS and SNS activity, it can provide a more complete view of autonomic flexibility and thus more sensitively reflect ANS changes associated with depression. While CAB indicates reciprocal balance between PNS and SNS activity (i.e., reciprocal SNS state is characterized by SNS activation combined with PNS withdrawal, and reciprocal PNS state is characterized by PNS activation combined with SNS withdrawal), CAR reflects overall ANS activity (i.e., activity of both PNS and SNS branches) [10]. In this context, our findings revealed reduced CAB at rest in adolescent major depression, which, together with the findings of shortened mean R-R intervals and LVET associated with higher –zLVET and lower zHF-HRV associated with reduced BRS, indicate a baseline reciprocal SNS state in adolescent major depression. These findings of abnormal cardiac autonomic functioning in adolescent MDD patients are consistent with those in young adults with depression [13]. Several mechanisms are suggested.

Consistent with the neurovisceral integration model and polyvagal theory [41,42], neural control of cardiac functioning mirrors a complex neurophysiological cortical-subcortical regulatory network, in which cortical areas allow the interpretation of safety and threat, and subcortical brainstem areas regulate the autonomic nervous system [43]. From this aspect, the inhibitory function of the prefrontal cortex is especially highlighted. Briefly, the prefrontal cortex inhibits subcortical regulatory centers, allowing the organism to adjust emotional, behavioral, and health-related processes [44,45]. Further, dynamic functional connectivity between the ventromedial prefrontal cortex and the amygdala is correlated with vagally-mediated heart rate variability (HF-HRV), and thus inhibitory control of the prefrontal cortex over limbic regions is closely related to changes in cardiac parasympathetic regulation [46]. In this vein, the prefrontal areas of the brain are hypoactive in depressed patients [47]. We assume that the disrupted inhibitory prefrontal function on subcortical sympatho-excitatory centers can result in different reciprocal sympathetic and parasympathetic activity indexed by lower CAB in adolescent MDD.

Given the independence of both ANS subsystems, it is valuable to examine how stress responses might influence the different relations between the two branches of ANS [10,15]. In this context, this study first explored the SNS and PNS coupling during complex stress responses (i.e., rest—mental stress (Go/NoGo task), and recovery (period after stress)) in MDD at adolescent age. Similar to the resting state, our findings showed that the lower CAB in association with shortened mean R-R intervals and LVET, higher parameters (–zLVET and HF-SBPV), lower lnHF-HRV, BRS, and zHF-HRV indicate SNS predominance associated with vagal withdrawal during the Go/NoGo task in adolescent major depression. On the other hand, no significant differences in CAB were found when compared to baseline and stress response. This result is in contrast with the findings of Bylsma et al. [12], who reported increased CAB from baseline for physiological (i.e., handgrip) and psychological (i.e., unsolvable puzzle) tasks, reflecting a shift to parasympathetic activation and/or sympathetic withdrawal in youth with a history of juvenile-onset depression [12]. These differences could be explained by the mutual influences of PNS-SNS coupling activities. Specifically for CAB, the space for sympathetic and parasympathetic reactivity to stress is constrained by the resting PNS and SNS activities that jointly define CAB. Consequently, the sympathetic and parasympathetic responses to stress and after stress are perpetually modifying the resting tone. Therefore, the inability to recover resting autonomic activity may constrain space for future reactivity [48]. In this context, decreased CAB in association with shortened mean R-R intervals, LVET, and higher –zLVET combined with decreased lnHF-HRV and BRS also persisted during the recovery phase in MDD adolescents compared to controls. Thus, we can assume a complex mutual influence of CAB by individual phases of the stress response: according to the “law of initial values” [49], resting PNS and SNS activities affect their values during stress, and, vice versa, the stress-related ANS activity can affect the resting PNS and SNS activities in adolescent MDD. Further, the variability in PNS-SNS coupling to an acute stressor depends on the type of stressor. In contrast to physiological stressors such as orthostatic tests evoking a uniform autonomic response (i.e., sympathetic activation associated with parasympathetic withdrawal), ANS activity during mental stress is influenced by cortical as well as subcortical regulatory centers, resulting in a higher variety of individual autonomic responses from both autonomic branches [10,11,12]. Therefore, we can assume a complex interplay of neurophysiological and psychological factors contributing to differences in PNS-SNS coupling in response to mental stressors (i.e., the Go/NoGo task) in adolescent depressed patients. With respect to CAR, the only difference between MDD and control groups was observed during the recovery phase, which, however, appeared to be driven by changes in the parameter LVET rather than PNS and SNS coactivation. In this way, the cardiac SNS dominance indicated by shortened LVET (and increased –zLVET) during the recovery phase can point to the slow return of the sympathetic activity to the baseline state following a stressor in adolescent MDD [10].

Further, this study, for the first time, assessed the sex effect on cardiac autonomic control using composite indices in adolescent depression. Specifically, the autonomic pattern of lower CAB along with reduced zHF-HRV and higher –zLVET was clearly observed only in the group of depressed boys, not in the group of depressed girls. Thus, MDD male patients contributed most to the overall difference in cardiac autonomic control between MDD patients and controls in our study. Moreover, based on our results, the parameter lnHF-SBPV was the sole sensitive index revealing the difference in the autonomic pattern between boys and girls (i.e., significantly increased lnHF-SBPV in boys) only in the control group during the recovery period. This sex-related effect has not been seen between boys and girls in the MDD group. However, in this context, it is also important to note that the parameter lnHF-SBPV has been significantly increased only in the group of depressed girls, not depressed boys, when compared to controls, likely explaining the disappearance of the sex-related difference in the MDD group. The importance of sex is highlighted in terms of the differential neuropsychological mechanisms underlying the relationship between cardiac autonomic control and depression [18]. First, depression can be conceptualized as a threat-related response due to enhanced neural responses to social threat signals [18,50]. In line with neurovisceral integration theory, the higher HF-HRV may represent greater prefrontal cortex inhibition in subcortical areas [42,51]. More specifically, greater activation in prefrontal regions could compensate for the increased activity of the amygdala, a brain area implicated in threat processing, which is potentially mediated by depression [2,52]. Thus, higher cardiovagal autonomic modulation reflecting increased prefrontal inhibition may represent greater self-regulatory reserves built up to deal with heightened depressive symptoms in females [14,18]. In line with psychophysiological stress theories, males tend to respond to stress by a “fight-or-flight” reaction contributing to high arousal and sympathetic active dominance, while females respond by “tend-and-befriend” associated rather with a calming state and vagal activation [18]. Second, a shift from lower vagal activity (pre-pubertal) to greater vagal activity (post-pubertal) in girls compared to boys may be related to hormonal changes during the sensitive adolescent period [53]. The effects of hormones such as estrogen leading to acetylcholine sensitivity, increased vagal tone, and/or different cortical development during adolescence compared to adulthood associated with vagal outflow in female adolescents are also considered potential mechanisms of sex differences [2,53,54,55]. Moreover, higher cardiac parasympathetic activity may serve as compensation for increased sympathetic activity in women, thereby maintaining optimal regulation of the periphery [2,56]. It is a question of whether sex differences in adolescence rather represent a “pre-stage” of MDD-linked compensatory responses manifested in adulthood or whether they only reflect hormonal and other physiological-related changes in cardiac autonomic regulation in vulnerable adolescent periods.

Lastly, the systemic effects of the ovarian cycle throughout the body play an important role, as females have been shown to experience different resting conditions and stress responses compared to their male counterparts. These sex-related differences can result from hormonal (mainly oestrogen and progesterone) fluctuations during the cycle, which trigger changes in all body systems, including the autonomic nervous system (ANS) [57,58]. Generally, oestrogen modulates ANS activity through an increase in parasympathetic nervous system activity and a decrease in sympathetic nervous system activity, while progesterone appears to act oppositely via increasing sympathetic drive [59,60]. It is important to note that oestrogen levels rise during the mid-follicular and mid-luteal phases, with a precipitous decrease after ovulation and at the end of the menstrual cycle. In contrast, progesterone levels rise after ovulation and during the luteal phase and decrease at the end of the menstrual cycle [61]. Concerning cardiovascular autonomic modulation, cardiac vagal autonomic modulatory activity is dominant in the follicular phase, and this effect decreases from the follicular to the luteal phase [62]. More specifically, HRV is increased prior to ovulation and then decreases until the new menses onset [59]. Further, while the cardiovagal BRS has been shown not to be affected by the individual phases of the cycle [63], the sympathetic vasomotor-mediated BRS has been reported to be higher in the mid-luteal phase (i.e., when oestrogen and progesterone levels are elevated) compared to the early follicular phase (i.e., when hormone levels are relatively low) [63,64,65]. With respect to vessels, both hormones (oestrogen and progesterone) can potentially modulate vascular regulation through their powerful vasodilatory effects [66,67]. To sum up, as the levels of both hormones—oestrogen and progesterone—fluctuate through the menstrual cycle and as both hormones can modulate autonomic-mediated cardiac and vascular regulation, it should be considered in the studies’ design to examine females within the same stage of the ovarian cycle.

### Limitations of Study

This study includes a relatively homogenous group of adolescent patients with MDD without comorbidities or pharmacotherapy; however, future research is needed to expand the sample size with respect to sex. Further, the smoking state, physical activity, or menstrual cycle phase potentially affecting cardiac autonomic regulation were not monitored. This study explored autonomic responses only to neuropsychological Go/NoGo tasks using cardiac-linked measures; therefore, future research is needed to study other effectors’ autonomic responses to different psychological or physiological stressors in adolescent MDD. Lastly, a six-minute recovery phase could possibly not be enough for SNS to return to baseline conditions.

## 5. Conclusions

Our study points to CAB as a promising sensitive biomarker for psychophysiological alterations associated with depression already at adolescent age, predominantly in adolescent depressed boys. Moreover, CAB can also be considered a sensitive marker to detect cardiac autonomic abnormalities in adolescent depression. Therefore, our findings could contribute to a better understanding of the involved mechanisms linking cardiac autonomic dysregulation and increased cardiovascular risk in adolescent MDD and lead to more personalized prevention of possible later cardiovascular diseases and consequently better life quality in adulthood.

## Figures and Tables

**Table 1:** Basic characteristics of the control and MDD groups.

Evaluated Parameter	Controls			MDD			*p*-Value				
	Total ^a^ (N = 80)	Girls ^b^ (N = 51)	Boys ^c^ (N = 29)	Total ^d^ (N = 85)	Girls ^e^ (N = 52)	Boys ^f^ (N = 33)	a vs. d	b vs. e	c vs. f	b vs. c	e vs. f
Age (years)	15.9 ± 0.15	16.0 ± 0.19	15.9 ± 0.27	15.7 ± 0.14	15.6 ± 0.18	15.9 ± 0.2	0.421	0.999	0.999	0.999	0.999
BMI (kg/m^2^)	21.5 ± 0.4	21.4 ± 0.5	21.8 ± 0.7	20.4 ± 0.2	20.7 ± 0.3	20.1 ± 0.4	0.007	0.999	0.091	0.999	0.996
WHR	0.84 ± 0.005	0.84 ± 0.007	0.84 ± 0.010	0.83 ± 0.004	0.83 ± 0.004	0.83 ± 0.006	0.064	0.847	0.999	0.999	0.994
CDI	6.7 ± 0.5	7.2 ± 0.6	6.0 ± 0.8	24.6 ± 0.9	23.5 ± 1.1	26.5 ± 1.4	<0.001	<0.001	<0.001	0.999	0.301

MDD—major depressive disorder, BMI—body mass index, WHR—waist to hip ratio, CDI—Children’s Depression Inventory. Data are expressed as mean ± SEM. a—total control group, b—control Girls group, c—control Boys group, d—total MDD group, e—MDD Girls group, f—control Boys group. The *p*-value a vs. d expresses the comparison between total control group and total MDD group. The *p*-value b vs. e expresses the comparison between control Girls group and MDD Girls group. The *p*-value c vs. f expresses the comparison between control Boys group and MDD Boys group. The *p*-value b vs. c expresses the comparison between control Boys group and control Girls group. The *p*-value e vs. f expresses the comparison between MDD Girls group and MDD Boys group. The results are considered statistically significant if *p* < 0.05.

**Table 2:** Cardiac autonomic parameters in the control and MDD groups.

Evaluated Parameter	Controls			MDD			*p*-Value				
	Total ^a^ (N = 80)	Girls ^b^ (N = 51)	Boys ^c^ (N = 29)	Total ^d^ (N = 85)	Girls ^e^ (N = 52)	Boys ^f^ (N = 33)	a vs. d	b vs. e	c vs. f	b vs. c	e vs. f
Baseline											
R-R intervals (ms)	799.0 ± 11.5	800.0 ± 11.5	798.0 ± 11.8	686 ± 10.2	692.0 ± 9.3	677.0 ± 11.6	<0.001	<0.001	0.010	0.999	0.999
lnHF-HRV (ms^2^)	2.04 ± 0.48	2.06 ± 0.49	1.99 ± 0.46	1.62 ± 0.49	1.71 ± 0.42	1.48 ± 0.57	0.282	0.999	0.999	0.999	0.999
LVET (ms)	292 ± 17.5	294.0 ± 18.2	287.0 ± 15.2	275 ± 20.4	278.0 ± 19.2	269.0 ± 21.2	<0.001	<0.001	0.006	0.999	0.999
SBP (mmHg)	113.0 ± 17.0	115.0 ± 16.4	110 ± 13.8	111.0 ± 17.0	108.0 ± 16.3	115.0 ± 15.5	0.999	0.917	0.994	0.978	0.993
DBP (mmHg)	69.9 ± 1.0	15.1 ± 1.0	14.8 ± 1.5	69.5 ± 1.2	67.3 ± 1.5	73.2 ± 2.1	0.999	0.999	0.999	0.999	0.999
BRS (ms/mmHg)	15.00 ± 0.84	15.10 ± 1.03	14.80 ± 1.46	9.86 ± 0.61	10.10 ± 0.69	9.56 ± 1.12	<0.001	0.011	0.149	0.999	0.999
lnHF-SBPV (mmHg^2^)	6.03 ± 0.03	5.99 ± 0.03	6.09 ± 0.05	6.20 ± 0.04	6.18 ± 0.05	6.22 ± 0.06	0.999	0.005	0.114	0.123	0.287
zHF-HRV	0.407 ± 0.101	0.453 ± 0.129	0.327 ± 0.163	−0.383 ± 0.102	−0.211 ± 0.111	−0.655 ± 0.189	<0.001	0.060	0.017	0.999	0.999
–zLVET	−0.252 ± 0.198	−0.283 ± 0.303	−0.198 ± 0.135	0.397 ± 0.106	0.231 ± 0.128	0.659 ± 0.177	0.032	0.999	0.988	0.999	0.999
CAB	0.659 ± 0.226	0.736 ± 0.324	0.524 ± 0.257	−0.780 ± 0.181	−0.441 ± 0.204	−1.310 ± 0.321	<0.001	0.061	0.005	0.999	0.999
CAR	0.155 ± 0.219	0.170 ± 0.334	0.129 ± 0.154	0.014 ± 0.102	0.020 ± 0.126	0.004 ± 0.176	0.999	0.999	0.999	0.999	0.999
Go/NoGo task											
R-R intervals (ms)	793 ± 14.5	781 ± 12.5	815 ± 15.6	698 ± 10.9	700 ± 9.6	694 ± 13.0	<0.001	0.035	<0.001	0.999	0.999
lnHF-HRV (ms^2^)	5.9 ± 1.61	5.68 ± 1.32	6.27 ± 1.97	4.66 ± 1.26	4.77 ± 1.08	4.47 ± 1.51	<0.001	0.010	<0.001	0.999	0.999
LVET (ms)	294 ± 15.9	296 ± 16.6	291 ± 14.4	276 ± 19.6	280 ± 16.9	270 ± 22	<0.001	0.003	<0.001	0.999	0.584
SBP (mmHg)	127.0 ± 20.2	128.0 ± 20.3	125.0 ± 17.6	122.0 ± 20.0	118.0 ± 17.6	129.0 ± 16.9	0.999	0.187	0.999	0.999	0.645
DBP (mmHg)	77.4 ± 1.28	77.7 ± 1.6	76.9 ± 2.1	76.0 ± 1.57	72.3 ± 1.8	82.2 ± 2.6	0.999	0.999	0.999	0.999	0.093
BRS (ms/mmHg)	16.80 ± 0.87	16.60 ± 1.04	17.40 ± 1.56	11.00 ± 0.70	11.00 ± 0.80	11.00 ± 1.29	<0.001	0.004	0.012	0.999	0.999
lnHF-SBPV (mmHg^2^)	13.70 ± 0.08	13.60 ± 0.09	13.70 ± 0.14	14.00 ± 0.08	14.10 ± 0.10	14.00 ± 0.16	0.020	0.001	0.279	0.329	0.758
zHF-HRV	0.098 ± 0.138	−0.011 ± 0.154	0.278 ± 0.262	−0.390 ± 0.078	−0.324 ± 0.085	−0.494 ± 0.149	<0.001	0.999	0.260	0.999	0.999
–zLVET	−0.457 ± 0.089	−0.545 ± 0.117	−0.301 ± 0.134	0.598 ± 0.194	0.232 ± 0.118	1.170 ± 0.451	<0.001	0.291	0.002	0.999	0.152
CAB	0.557 ± 0.179	0.544 ± 0.218	0.578 ± 0.313	−0.998 ± 0.228	−0.557 ± 0.173	−1.670 ± 0.504	<0.001	0.169	<0.001	0.999	0.403
CAR	−0.362 ± 0.150	−0.566 ± 0.169	−0.023 ± 0.275	0.208 ± 0.188	−0.092 ± 0.110	0.680 ± 0.444	0.434	0.999	0.999	0.999	0.999
Recovery											
R-R intervals (ms)	782 ± 12.6	785 ± 13.6	776 ± 10.6	695 ± 10.5	706 ± 9.8	678 ± 11.4	<0.001	0.063	0.101	0.999	0.999
lnHF-HRV (ms^2^)	5.40 ± 1.29	5.44 ± 1.30	5.34 ± 1.29	4.73 ± 1.37	4.73 ± 1.02	4.73 ± 1.80	0.014	0.311	0.999	0.999	0.999
LVET (ms)	292 ± 17.4	295 ± 18.4	286 ± 13.9	275 ± 19.5	280 ± 16.8	267 ± 21.5	<0.001	0.003	0.004	0.999	0.170
SBP (mmHg)	119.0 ± 19.9	121.0 ± 17.5	116.0 ± 16.3	117.0 ± 19.9	114.0 ± 17.4	122.0 ± 16.2	0.999	0.819	0.997	0.991	0.935
DBP (mmHg)	73.5 ± 1.22	74.1 ± 1.5	72.5 ± 2.2	74.4 ± 1.54	71.3 ± 1.9	79.3 ± 2.4	0.999	0.999	0.999	0.999	0.999
BRS (ms/mmHg)	15.10 ± 0.76	15.10 ± 0.97	14.90 ± 1.23	9.53 ± 0.46	10.20 ± 0.59	8.51 ± 0.72	<0.001	0.045	0.028	0.999	0.999
lnHF-SBPV (mmHg^2^)	14.20 ± 0.09	14.00 ± 0.09	14.50 ± 0.18	14.50 ± 0.12	14.40 ± 0.14	14.70 ± 0.19	0.085	0.033	0.410	0.009	0.111
zHF-HRV	−0.033 ± 0.155	0.054 ± 0.184	−0.186 ± 0.279	−0.229 ± 0.108	−0.224 ± 0.104	−0.232 ± 0.229	0.999	0.999	0.999	0.999	0.999
–zLVET	−0.431 ± 0.096	−0.598 ± 0.126	−0.138 ± 0.127	0.569 ± 0.189	0.182 ± 0.115	1.180 ± 0.436	<0.001	0.286	0.013	0.999	0.081
CAB	0.398 ± 0.193	0.652 ± 0.235	−0.048 ± 0.322	−0.798 ± 0.196	−0.409 ± 0.183	−1.410 ± 0.396	<0.001	0.262	0.217	0.999	0.872
CAR	−0.464 ± 0.170	−0.544 ± 0.211	−0.324 ± 0.291	0.340 ± 0.238	−0.046 ± 0.118	0.949 ± 0.574	0.011	0.999	0.181	0.999	0.486

MDD—major depressive disorders, lnHF-HRV—spectral power in the high-frequency band of the heart rate variability, LVET—left ventricular ejection time, SBP—systolic blood pressure, DBP—diastolic blood pressure, BRS—baroreflex sensitivity, lnHF-SBPV—spectral power in the high-frequency band of the systolic blood pressure variability, zHF-HRV—z score of spectral power in the high-frequency band of the heart rate variability, –zLVET—minus z score of left ventricular ejection time, CAB—cardiac autonomic balance, CAR—cardiac autonomic regulation. All data are expressed as mean ± SEM. a—total control group, b—control Girls group, c—control Boys group, d—total MDD group, e—MDD Girls group, f—control Boys group. The *p*-value a vs. d expresses the comparison between total control group and total MDD group. The *p*-value b vs. e expresses the comparison between control Girls group and MDD Girls group. The *p*-value c vs. f expresses the comparison between control Boys group and MDD Boys group. The *p*-value b vs. c expresses the comparison between control Boys group and control Girls group. The *p*-value e vs. f expresses the comparison between MDD Girls group and MDD Boys group. The results are considered statistically significant if *p* < 0.05.

## Data Availability

Data are available upon reasonable request from the corresponding author.
